# A limited sampling procedure for estimating adriamycin pharmacokinetics in cancer patients.

**DOI:** 10.1038/bjc.1989.226

**Published:** 1989-07

**Authors:** M. C. Launay, G. Milano, A. Iliadis, M. Frenay, N. Namer

**Affiliations:** Laboratoire de PharmacocinÃ©tique, INSERM U 278, FacultÃ© de Pharmacie, Marseille, France.

## Abstract

The aim of this study was to find a procedure allowing estimation of individual pharmacokinetic parameters for adriamycin with minimal cost and disturbance for the patient. Twenty-five patients with breast cancer were treated by short infusion of adriamycin at a dose of 12 mg m-2 week-1 (41 courses). Population characteristics were determined on 15 randomly chosen courses (10 patients, group I) in order to define two optimal sampling times (26 min and 24 h) and to perform Bayesian estimation on the remaining 26 courses (17 patients, group II). For patients of group II, Bayesian estimation (BE) associated with a reduced sub-optimal sampling protocol (20 min and 24 h) was compared with maximum likelihood estimation (MLE), the classical procedure. Regression analysis of clearance values obtained after BE versus MLE indicated a high correlation coefficient (r = 0.969) with the slope (a = 0.991 +/- 0.085) and the intercept (b = 2.271 +/- 4.810) close to 1 and 0 respectively. This original method is thus valid to measure accurately adriamycin clearance; it improves patient comfort and can be used routinely.


Br. J. Cancer (1989), 60, 89-92 © The Macmillan Press Ltd., 1989

### A limited sampling procedure for estimating adriamycin pharmacokinetics in cancer patients

M.C. Launay[1], G. Milano[2], A. Iliadis[1], M. Frenay[2] & N. Namer[2]

[1]*Laboratoire de Pharmacocinétique, INSERM U 278, Faculté de Pharmacie, 27 Bd J. Moulin, 13385 Marseille Cedex 5, France; and* [2]*Centre Antoine-Lacassagne, 36 Av Voie-Romaine, 06054 Nice Cedex, France.*

**Summary** The aim of this study was to find a procedure allowing estimation of individual pharmacokinetic parameters for adriamycin with minimal cost and disturbance for the patient. Twenty-five patients with breast cancer were treated by short infusion of adriamycin at a dose of $12\,mg\,m^{-2}\,week^{-1}$ (41 courses). Population characteristics were determined on 15 randomly chosen courses (10 patients, group I) in order to define two optimal sampling times (26 min and 24 h) and to perform Bayesian estimation on the remaining 26 courses (17 patients, group II). For patients of group II, Bayesian estimation (BE) associated with a reduced sub-optimal sampling protocol (20 min and 24 h) was compared with maximum likelihood estimation (MLE), the classical procedure. Regression analysis of clearance values obtained after BE versus MLE indicated a high correlation coefficient ($r = 0.969$) with the slope ($a = 0.991 \pm 0.085$) and the intercept ($b = 2.271 \pm 4.810$) close to 1 and 0 respectively. This original method is thus valid to measure accurately adriamycin clearance; it improves patient comfort and can be used routinely.

Pharmacokinetic population studies have been developed in recent years to describe intra- and interindividual variabilities (Sheiner & Beal, 1981*a*). Among the available methods, Bayesian estimation (BE) allows satisfactory model parameter estimation with a limited number of sampling points and thus reduces hospitalisation time as well as cost of pharmacokinetic studies (Sheiner *et al.*, 1979). Such methodological schemes associating BE with a population study have been performed for several drugs (D'Argenio & Khakmahd, 1983; Lacarelle *et al.*, 1987; Vozeh & Steiner, 1987; Serre-Debeauvais *et al.*, 1987) but few anticancer ones (Iliadis *et al.*, 1985; Favre *et al.*, 1987). Moreover, sampling points are generally chosen arbitrarily or sometimes by using more sophisticated approaches like sensitivity functions or stepwise linear regression (Ratain & Vogelzang, 1987). But these approaches are not optimal, since they do not provide maximal precision in model parameters.

Adriamycin (ADM) is a broad-spectrum antineoplastic drug which is active against a wide variety of tumours. Experimental studies have suggested that ADM concentration-time product (area under the curve, AUC) is a determinant factor for tumour cell lethality (Eichholtz-Wirth, 1980; Ritch *et al.*, 1982). Robert *et al.* (1982) observed a correlation between pharmacokinetic parameters and clinical short-term response. Finally, according to Powis (1985), AUC is the best pharmacokinetic parameter for predicting anticancer pharmacodynamic events. As well as the relationships between AUC and response, there is also pharmacokinetic variability. For example, Rodvold *et al.* (1988) reported a reduced systemic clearance (Cl) of ADM in the obese. It is thus necessary to control this variability when dosage regimen calculations are required. Nevertheless, for breast cancer patients receiving regular ADM administrations, it is difficult to determine AUC or Cl (Cl = dose AUC) on the basis of the entire concentration-time curve as this requires a prolonged hospitalisation time and several blood samples. We applied a method of limited sampling associated with BE to ADM pharmacokinetics. The study was undertaken in a homogeneous group of 25 patients with breast cancer receiving several courses of ADM (a total of 41 courses). It consisted of two complementary steps: 15 courses were used to determine population characteristics and the optimal, limited sampling protocol (two points); the remaining 26 were considered to validate the method. This was done by comparing Cl values computed by using classical maximum likelihood estimation (MLE) and those computed by BE with the limited sampling protocol.

Correspondence: M.C. Launay.
Received 24 October 1988, and accepted in revised form 22 February 1989.

### Materials and methods

### *Patients*

Twenty-five patients with advanced breast cancer (all women; mean age 67.5 years, range 41-85) were treated by ADM on the basis of weekly injections ($12\,mg\,m^{-2}\,week^{-1}$). Included were 42% of patients with primary tumour only, 10% with primary and distal metastases, 38% with multifocal metastases and 10% with isolated metastases (liver and bone). There was no concomitant irradiation. Cumulative dose of ADM per patient was (mean, range) 372 mg, 100-600 mg. Five patients had previously been treated by a chemotherapy regimen including anthracyclines and the total cumulative dose, including weekly ADM, was (mean, range) 640 mg, 160-1,011 mg; two other patients had received a chemotherapy protocol without anthracyclines. All patients had normal pretreatment serum bilirubin ($5-17\,\mu mol\,l^{-1}$). In all cases, ADM was given by a 5-min infusion through a venous catheter. A complete pharmacokinetic study concerning ADM given on a weekly schedule on the same patients has been published (Frenay *et al.*, 1988).

### *Analytical method*

A complete pharmacokinetic profile was obtained for the first injection in 25 patients and once a month in 10 of them, i.e. in most cases at the 5th, 9th and 13th injections. Blood samples were drawn 5, 20, 40 min and 1, 2, 4, 8 and 24 h after the start of injection. As all patients were treated on an outpatient basis, it was not possible to get blood samples for more than the 24 h. From these patients and their cycles, 41 concentration-time curves were obtained. Blood samples were collected in EDTA tubes and immediately centrifuged. Plasma was stored at $-20^{\circ}C$ until analysis (within 2 weeks). ADM was quantified by HPLC. Extraction was performed on Sep-Pak C18 cartridges (Millipore, Waters) as previously described (Robert, 1980) with slight modifications: cartridges were conditioned by successive elutions with 2 ml methanol and 5 ml phosphate buffer ($Na_2HPO_4$, 0.05 M; $NaH_2PO_4$, 0.05 M; 2:1). One ml of plasma (patient or plasma for standard curve) spiked with 50 $\mu l$ of internal standard (daunorubicin, $1\,nmol\,ml^{-1}$) was passed through the cartridge, followed by 1 ml of phosphate buffer (discarded). Drug material was eluted by 3 ml of methanol in previously siliconated tubes. After drying ($N_2$, 40°C), the residue was diluted in 250 μl of HPLC buffer, centrifuged for 10 min at 4°C, and injected into the HPLC system. Analysis was performed on an HPLC column μBondapak phenyl 30 mm × 0.4 mm ID (Millipore, Waters) with a $CH_3CN$-formiate buffer (33.5/66.5), pH 4, at a flow rate of 2.5 ml min$^{-1}$. Fluorescent detection was performed with a spectrofluorimeter (Kontron SFM 25) at $\lambda_{ex} = 470$ nm and $\lambda_{em} = 600$ nm.

### *Pharmacokinetic study*

By using MLE, all concentration–time curves were fitted to an open two-compartment model expressed as a sum of exponentials:

$$c(t) = A \, . \, e^{-at} + B \, . \, e^{-bt}$$

where $A$ and $B$ (the coefficients) and $a$ and $b$ (the exponents) are the model macroconstants allowing calculation of concentration $c$ at time $t$. These macroconstants, evaluated by MLE, constitute the reference values. Note that MLE is the criterion defined by minimising the sum of squares of the concentrations weighted by the model predictions, assuming normality for measurement error with variance proportional to the squared expected concentration value. This procedure is sometimes called iteratively reweighted least squares (Sheiner & Beal, 1985). The initial set of 41 cycles (25 patients) was randomly divided into two groups: one including 15 cycles (10 patients, group I) was used to calculate population characteristics by the standard two-stage method (Sheiner & Beal, 1981*a*). Inter- and intra-individual variabilities are both present in population studies; in order to express the former, we randomly chose 10 subjects and, to express the latter, five additional courses were drawn in four of these 10 subjects. This was done to take into account changes in the pharmacokinetic behaviour between two successive courses, e.g. a possible time-dependence. The other group of 26 cycles (17 subjects, group II) was used as a test data set to evaluate the performance of BE associated with a reduced sampling protocol.

First, population data allowed computation of an optimal sampling time set to be used for all patients of group II. For ethical reasons and improved patient comfort, we planned only two sampling points, at least 5 min apart, during the 24 h following the ADM administration. This calculation was performed on the basis of D-optimality theory. Its main characteristic is to guarantee a precise estimation of the model pharmacokinetic parameters (the macroconstants) by optimising a non-linear cost function (Bard, 1974; Launay & Iliadis, 1988). The criterion value, i.e. the maximised cost function value, is inversely proportional to the volume of the confidence region for the parameter estimates (Atkinson & Hunter, 1968); the higher its value is, the smaller the volume is and the more precise the estimates are.

In fact, the set of two optimal sampling times yielded by the D-optimality criterion remains theoretical because these times differ from the experimental ones. Thus, to choose the available experimental sampling times closest to the theoretical ones, we compared the D-optimality criterion values obtained for all experimental two time-point combinations.

Secondly, as population data are *a priori* information on the ADM pharmacokinetic behaviour, they are used in the BE criterion performed on the test data set (Iliadis *et al.*, 1985). All MLE and BE as well as optimal design calculations were performed on a desktop computer (Tektronix 4052) using APIS software (Iliadis, 1985; Launay & Iliadis, 1988). The parameters identified by MLE and BE are the macroconstants from which Cl is computed (Wagner, 1975). We selected Cl estimation since this parameter has the greatest potential for clinical applications. It is also the most useful parameter for the evaluation of an elimination mechanism.

### *Statistics*

Efficiency of BE with respect to MLE is studied on Cl. The performance of Cl prediction was analysed according to the suggestions of Sheiner & Beal (1981*b*). A correlation analysis was done to confirm the prediction reliability of Bayesian estimates with regard to maximum likelihood estimates.

### Results

ADM population data describing the mean behaviour (mean parameters), interindividual variability (covariance and correlation matrix) and residual intra-individual variability (coefficient of variation of residual variability) are presented in Table I. Because of the low residual variability (15%) and its measurement error (10%), two compartment modelling is a good description of the observed data. The coefficient of variation of the exponents ($a$ and $b$) was smaller than that of the coefficients ($A$ and $B$), indicating a low interindividual variability for the slopes of the two phases. The low correlation coefficients expressing covariances indicate that there is no preferential relation between parameters. Figure 1 shows the mean kinetic profile of ADM after a 20 mg bolus injection with its 68.3% confidence intervals expressing inter-individual variability of concentrations in function of time.

Design optimisation with population data provided the two 'theoretical' time points located at 26 min and 24 h, corresponding to a criterion value of 35.743. Computation of

**Table I** Population pharmacokinetic parameters evaluated for 15 patients (group I)

| | | *Macroconstants* | | | |
|---|---|---|---|---|---|
| | | $A$ | $B$ | $a$ | $b$ |
| Mean population parameters $\theta_0$ | | $7.058 \times 10^{-2}$ | $7.355 \times 10^{-4}$ | 8.665 | $5.589 \times 10^{-2}$ |
| Coefficient of variation (%) | | 44 | 51 | 32 | 27 |
| Covariance and correlation matrix | $A$ | $9.863.10^{-4}$ | 0.249 | 0.273 | 0.292 |
| | $B$ | $2.972.10^{-6}$ | $1.441.10^{-7}$ | −0.525 | 0.239 |
| | $a$ | $2.374.10^{-2}$ | $-5.518.10^{-4}$ | 7.659 | $-9.69.10^{-5}$ |
| | $b$ | $1.408.10^{-4}$ | $1.393.10^{-6}$ | $-4.112.10^{-6}$ | $2.35.10^{-4}$ |
| Coefficient of variation of residual variability = 15% | | | | | |

The macroconstants are the model parameters: $A$ and $B$ are the coefficients and $a$ and $b$ are the exponents. The covariance-correlation matrix is divided into three parts: the correlations between two parameters are in the upper triangle (1). They were computed from the covariances which are written in the lower triangle (2). The variance of each parameter (squared standard deviation) is presented on the diagonal of the matrix (3).

**Figure 1** Mean plasma adriamycin concentration–time curve for a 20 mg bolus injection. The confidence intervals are drawn at $P=0.683$.

the D-optimality criterion for all experimental two time-point combinations is summarised in Table II: the experimental value closest to 35.743, the maximum, was 35.585, corresponding to the 20 min and 24 h combination. The 20 min sampling time was the most informative one as the highest criterion values are in its column.

We therefore used the experimental sampling protocol of 20 min and 24 h to perform BE on the test data set (group II data). Estimated macroconstants then served to compute individual Cl values. The central question that arises was: is it possible to predict reliable Cl values from two sampling points, thus precluding the need for an entire concentration-time curve? Computing bias and precision of Cl values allowed evaluation of predictive performance of the Bayesian method. Their mean values on the test set were respectively 1.86 and 7.811 h$^{-1}$, with associated 97.5% confidence intervals of (−1.26, 4.98) and (5.19, 9.79) respectively. We concluded that bias was not significantly different from zero (its confidence interval includes zero) and the positive sign indicates that BE Cl is overestimated by about 1.861 h$^{-1}$. The overestimation is not significant. We noted also that precision of Cl estimates was low with respect to the dispersion of Cl values in the population: the standard deviation of BE Cl expressing interindividual variability, computed on group II data, reached 29.851 h$^{-1}$. Thus the ratio of this dispersion to the precision of BE Cl estimation is 0.262; this means that the Bayesian procedure is discriminative enough to distinguish subjects within members of the same population.

Figure 2 presents regression analysis of Cl values obtained after BE versus MLE ones. The correlation coefficient ($r=0.969$) is high and differs significantly from zero ($P<0.001$, d.f. = 25). The estimated values of the slope ($a=0.991\pm0.085$) and the intercept ($b=2.271\pm4.810$) are close to 1 and 0 respectively, showing the statistical equivalence between MLE and BE Cl values.

It is interesting to note that BE was able to predict clearance values included within two extremes of 6.575 and 137.451 h$^{-1}$ (a ratio of 1/20) with moderate relative errors of 21.2 and 9.9% respectively. Other than for the patient with the highest relative error of 76.8%, bias never exceeded 25.9%.

**Table II** D-optimality criterion value for all combinations between two experimental sampling times

| | *5 min* | *20 min* | *20 min* | *1 h* | *2 h* | *4 h* | *8 h* |
|---|---|---|---|---|---|---|---|
| 20 min | 34.933 | – | – | – | – | – | – |
| 40 min | 34.910 | 34.724 | – | – | – | – | – |
| 1 h | 34.362 | 35.112 | 34.193 | – | – | – | – |
| 2 h | 34.298 | 35.149 | 34.302 | 33.034 | – | – | – |
| 4 h | 34.296 | 35.140 | 34.296 | 33.060 | 32.972 | – | – |
| 8 h | 34.317 | 35.157 | 34.349 | 33.220 | 33.108 | 33.013 | – |
| 24 h | 34.648 | **35.585** | 35.059 | 34.315 | 34.196 | 34.067 | 33.802 |

The computed theoretical value of the D-optimality criterion, 35.743, was obtained for 26 min and 24 h.

**Figure 2** The difference between clearance values obtained by MLE and by BE ($r=0.969$, $P<0.001$).

### Discussion

The present study develops a complete procedure to efficiently estimate pharmacokinetic parameters with a minimal clinical cost. Its main contribution lies in the choice of a two-point sampling protocol guaranteeing a precise estimation of the model pharmacokinetic parameters. The good predictive performances reported indicate that Bayesian procedures should be developed for Cl prediction of ADM. The two time-point protocol (20 min, 24 h) proved efficient but a better estimation of the pharmacokinetic parameters should be obtained with 26 min and 24 h, the theoretically most informative sampling points, yielding more accurate values for new patients. In many clinical situations, determination of ADM Cl may be relevant. For example, in hepatic impairment, the relationship between the bilirubin level and the degree of alteration in ADM pharmacokinetics is not obvious (Kaye *et al.*, 1985). It is thus difficult to adjust the ADM dose with regard to bilirubin level for a given patient. The limited sampling procedure leads to easy estimation of ADM Cl and could be used for monitoring of patients with hepatic abnormalities. Besides, since ADM Cl is reduced in obese patients (Rovold *et al.*, 1988), its determination may be justified in this category of patients; a limited sampling procedure can be very useful as venous access is often difficult in obese patients. The method developed could also be of use for quicker estimations of AUC (Cl = dose/AUC), while reducing the difficulties of trials. Moreover, the two-point sampling protocol can be recalculated for periods longer than 24 h to take into account the part of the AUC due to a late elimination phase. In this case, the protocol still reduces the number of samplings while improving patient comfort. Another limited sampling model has recently been developed to estimate vinblastine AUC by using two time points selected by a stepwise multiple regression analysis (Ratain & Vogelzang, 1987). More recently, this team carried out the same kind of study with amonafide (Ratain *et al.*, 1988). In agreement with these authors, we think that a limited sampling procedure is of great clinical interest because it suppresses the need for continuous hospitalisation and minimises the number of venous punctions. This approach is based on calculation of population characteristics, thus showing the interest and need of interpatient variability studies. We think that a reduced cost should lead to a larger scale development of pharmacokinetic studies, a better understanding of anticancer pharmacodynamics and an optimisation of dosing.

### References

ATKINSON, A.C. & HUNTER, W.G. (1968). The design of experiments for parameter estimation. *Technometrics*, **10**, 271.

BARD, Y. (1974). Design criterion for parameter estimation. In *Nonlinear Parameter Estimation*, p. 262. Academic Press: New York and London.

D'ARGENIO, D.Z. & KHAKMAHD, K. (1983). Adaptive control of theophylline therapy: importance of blood sampling times. *J. Pharmacokinet. Biopharm.*, **11**, 547.

EICHHOLTZ-WIRTH, H. (1980). Dependence of the cytostatic effect of adriamycin on drug concentration and exposure time *in vitro*. *Br. J. Cancer*, **41**, 886.

FAVRE, R., CHARBIT, M., RINALDI, Y., ILIADIS, A., CARCASSONNE, Y. & CANO, J.P. (1987). Optimization of cisplatin (DDP) dosage regimen administered by continuous 5-day infusion using Bayesian estimation. *Proc. AACR*, **28**, 434.

FRENAY, M., MILANO, G., RINEE, N. and 4 others (1988). Pharmacokinetics of weekly low dose doxorubicin. *Eur. J. Cancer Clin. Oncol.* (in the press).

ILIADIS, A. (1985). APIS. A computer program for clinical pharmacokinetics. *J. Pharm. Clin.*, **4**, 573.

ILIADIS, A., BACHIR-RAHO, M., BRUNO, R. & FAVRE, R. (1985). Bayesian estimation and prediction of clearance in high-dose methotrexate infusions. *J. Pharmacokinet. Biopharm.*, **13**, 101.

KAYE, S.B., CUMMINGS, J. & KERR, D.J. (1985). How much does liver disease affect the pharmacokinetics of adriamycin? *Eur. J. Cancer Clin. Oncol.*, **21**, 893.

LACARELLE, B., GRANTHIL, C., MANELLI, J.C., BRUDER, N., FRANCOIS, G. & CANO, J.P. (1987). Evaluation of a Bayesian method of amikacin dosing in intensive care unit patients with normal or impaired renal function. *Ther. Drug. Monit.*, **7**, 258.

LAUNAY, M.C. & ILIADIS, A. (1988). A feasibility study of optimal sampling schedules in clinical pharmacokinetics. *Proc. IMACS*, **5**, 131.

POWIS, G. (1985). Anticancer drug pharmacodynamics. *Cancer Chemother. Pharmacol.*, **14**, 177.

RATAIN, M.J., STAUBUS, A.E., SCHILSKY, R.L. & MALPEIS, L. (1988). Limited sampling models for amonafide (NSC 308847) pharmacokinetics. *Cancer Res.*, **48**, 4127.

RATAIN, M.J. & VOGELZANG, N.J. (1987). Limited sampling model for vinblastine pharmacokinetics. *Cancer Treat. Rep.*, **71**, 935.

RITCH, P.S., OCCHIPINTI, S.J., SKRAMSTAD, K.S. & SHACKEY, S.E. (1982). Increased relative effectiveness of doxorubicin against slowly proliferating sarcoma 180 cells after prolonged drug exposure. *Cancer Treat. Rep.*, **66**, 1159.

ROBERT, J. (1980). Extraction of anthracyclines from biological fluids for HPLC evaluation. *J. Liquid Chromatogr.*, **3**, 1561.

ROBERT, J., ILIADIS, A., HOERNI, B., CANO, J.P., DURAND, M. & LAGARDE, C. (1982). Pharmacokinetics of adriamycin in patients with breast cancer: correlation between pharmacokinetic parameters and clinical short-term response. *Eur. J. Cancer. Clin. Oncol.*, **18**, 739.

ROVOLD, K.A., RUSHING, D.A. & TEWKSBURY, D.A. (1988). Doxorubicin clearance in the obese. *J. Clin. Oncol.*, **6**, 1321.

SERRE-DEBEAUVAIS, F., ILIADIS, A., TRANCHAND, B. and 5 others (1987). Bayesian estimation of cyclosporin clearance in bone marrow graft. *Proceedings of the Annual Meeting of Pharmacologists*, Nice.

SHEINER, L.B. & BEAL, S.L. (1981*a*). Evaluation of methods for estimating population pharmacokinetic parameters. II. Biexponential model and experimental pharmacokinetic data. *J. Pharmacokinet. Biopharm.*, **9**, 635.

SHEINER, L.B. & BEAL, S.L. (1981*b*). Some suggestions for measuring predictive performance. *J. Pharmacokinet. Biopharm.*, **9**, 503.

SHEINER, L.B. & BEAL, S.L. (1985). Pharmacokinetic parameter estimates from several Least Squares procedures: superiority of extended least squares. *J. Pharmacokinet. Biopharm.*, **13**, 185.

SHEINER, L.B., BEAL, S.L., ROSENBERG, B. & MARATHE, B.B. (1979). Forecasting individual pharmacokinetics. *Clin. Pharmacol. Ther.*, **26**, 294.

VOZEH, S. & STEINER, C. (1987). Estimates of the population pharmacokinetic parameters and performance of Bayesian feedback: a sensitivity analysis. *J. Pharmacokinet. Biopharm.*, **15**, 511.

WAGNER, J.G. (1975). *Fundamentals of Clinical Pharmacokinetics*. Drug Intelligence Publishers: Hamilton.


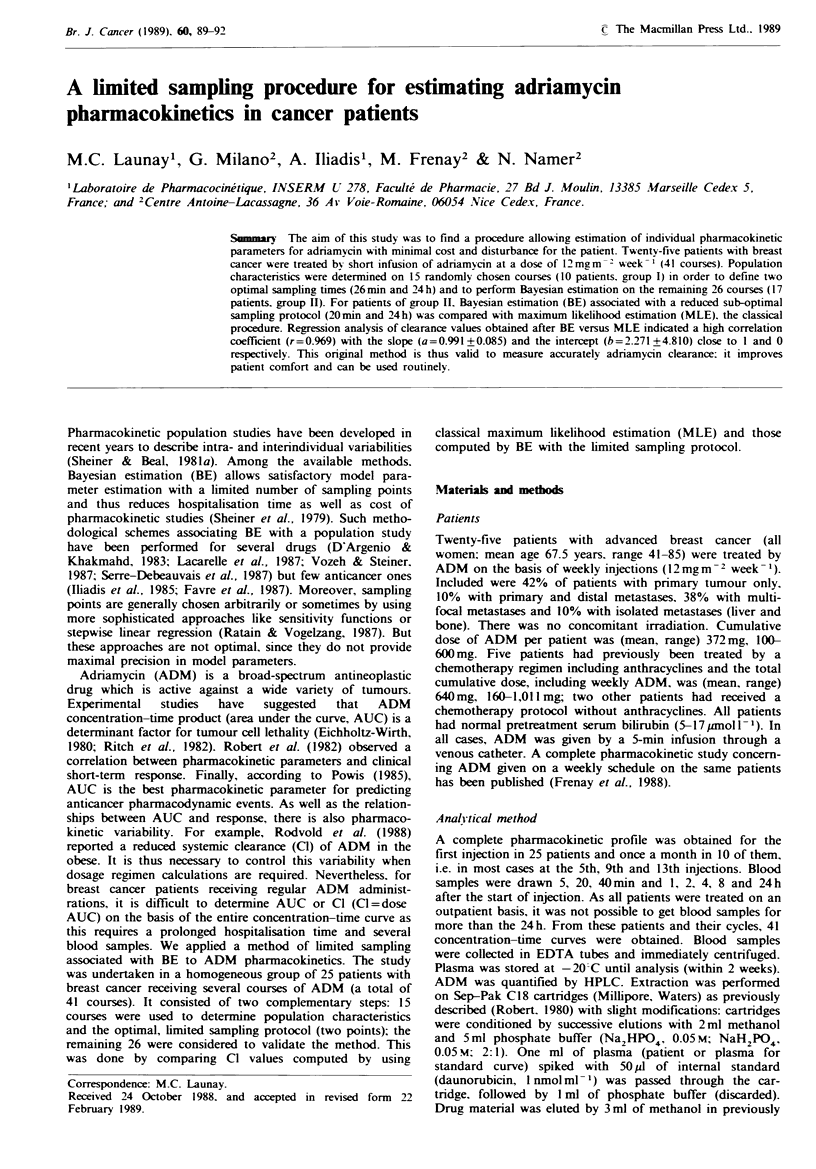

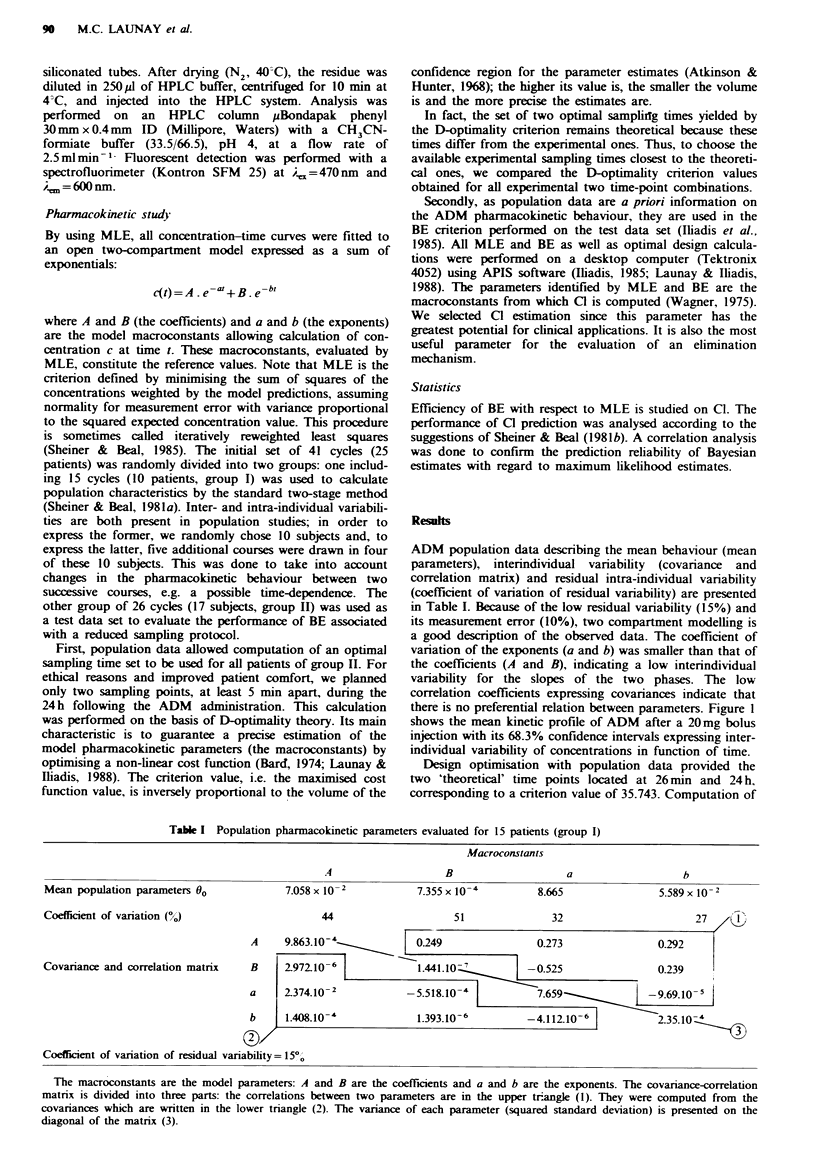

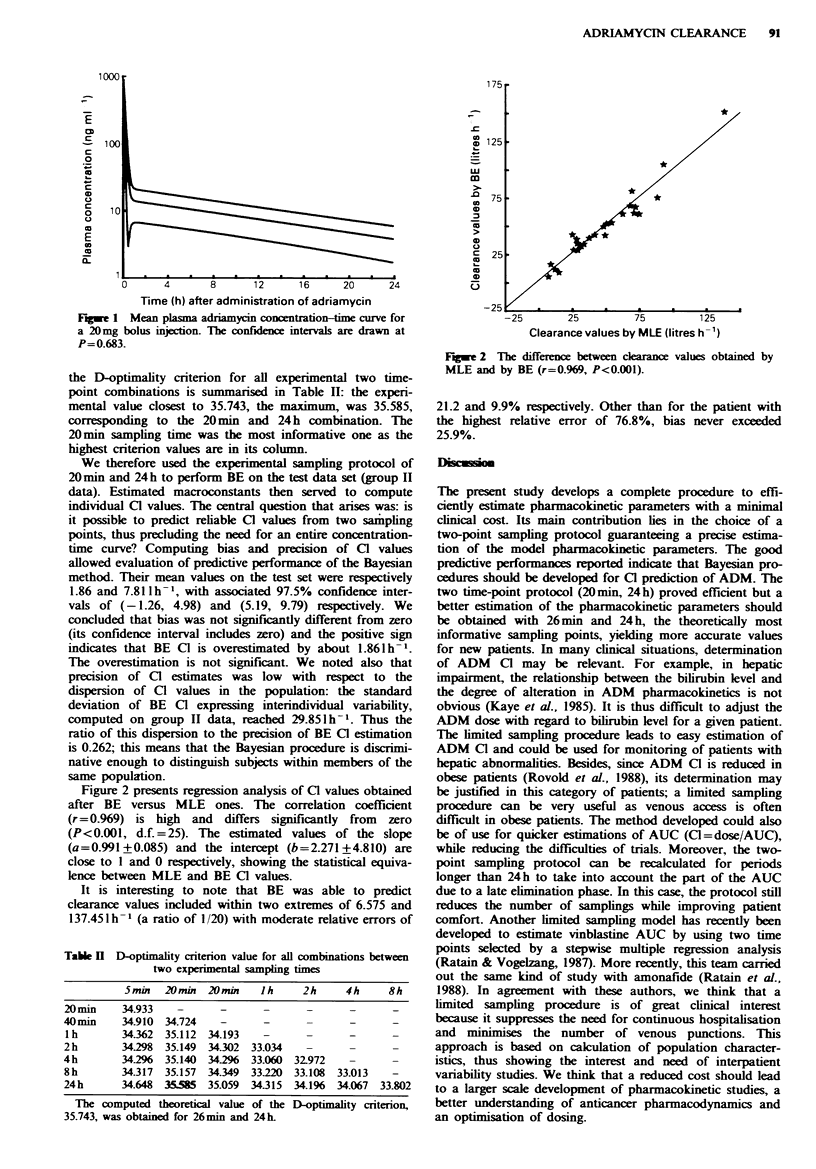

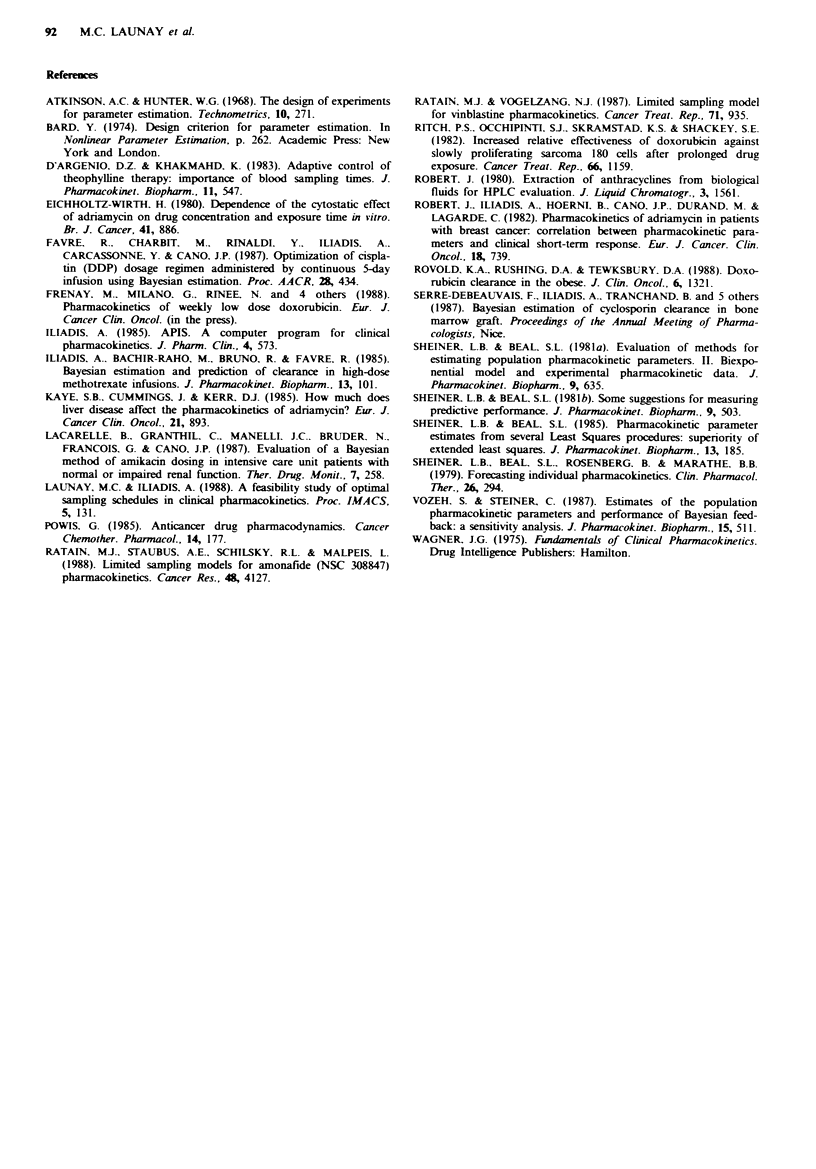


## References

[OCR_00487] D'Argenio D. Z., Khakmahd K. (1983). Adaptive control of theophylline therapy: importance of blood sampling times.. J Pharmacokinet Biopharm.

[OCR_00492] Eichholtz-Wirth H. (1980). Dependence of the cytostatic effect of adriamycin on drug concenration and exposure time in vitro.. Br J Cancer.

[OCR_00512] Iliadis A., Bachir-Raho M., Bruno R., Favre R. (1985). Bayesian estimation and prediction of clearance in high-dose methotrexate infusions.. J Pharmacokinet Biopharm.

[OCR_00517] Kaye S. B., Cummings J., Kerr D. J. (1985). How much does liver disease affect the pharmacokinetics of adriamycin?. Eur J Cancer Clin Oncol.

[OCR_00533] Powis G. (1985). Anticancer drug pharmacodynamics.. Cancer Chemother Pharmacol.

[OCR_00537] Ratain M. J., Staubus A. E., Schilsky R. L., Malspeis L. (1988). Limited sampling models for amonafide (NSC 308847) pharmacokinetics.. Cancer Res.

[OCR_00542] Ratain M. J., Vogelzang N. J. (1987). Limited sampling model for vinblastine pharmacokinetics.. Cancer Treat Rep.

[OCR_00546] Ritch P. S., Occhipinti S. J., Skramstad K. S., Shackney S. E. (1982). Increased relative effectiveness of doxorubicin against slowly proliferating sarcoma 180 cells after prolonged drug exposure.. Cancer Treat Rep.

[OCR_00558] Robert J., Illiadis A., Hoerni B., Cano J. P., Durand M., Lagarde C. (1982). Pharmacokinetics of adriamycin in patients with breast cancer: correlation between pharmacokinetic parameters and clinical short-term response.. Eur J Cancer Clin Oncol.

[OCR_00563] Rodvold K. A., Rushing D. A., Tewksbury D. A. (1988). Doxorubicin clearance in the obese.. J Clin Oncol.

[OCR_00573] Sheiner L. B., Beal S. L. (1981). Evaluation of methods for estimating population pharmacokinetic parameters. II. Biexponential model and experimental pharmacokinetic data.. J Pharmacokinet Biopharm.

[OCR_00583] Sheiner L. B., Beal S. L. (1985). Pharmacokinetic parameter estimates from several least squares procedures: superiority of extended least squares.. J Pharmacokinet Biopharm.

[OCR_00579] Sheiner L. B., Beal S. L. (1981). Some suggestions for measuring predictive performance.. J Pharmacokinet Biopharm.

[OCR_00588] Sheiner L. B., Beal S., Rosenberg B., Marathe V. V. (1979). Forecasting individual pharmacokinetics.. Clin Pharmacol Ther.

[OCR_00593] Vozeh S., Steiner C. (1987). Estimates of the population pharmacokinetic parameters and performance of Bayesian feedback: a sensitivity analysis.. J Pharmacokinet Biopharm.

